# Zoledronic Acid Produces Combinatory Anti-Tumor Effects with Cisplatin on Mesothelioma by Increasing p53 Expression Levels

**DOI:** 10.1371/journal.pone.0060297

**Published:** 2013-03-28

**Authors:** Shinya Okamoto, Yuanyuan Jiang, Kiyoko Kawamura, Masato Shingyoji, Toshihiko Fukamachi, Yuji Tada, Yuichi Takiguchi, Koichiro Tatsumi, Hideaki Shimada, Kenzo Hiroshima, Hiroshi Kobayashi, Masatoshi Tagawa

**Affiliations:** 1 Department of Biochemistry, Graduate School of Pharmaceutical Sciences, Chiba University, Chiba, Japan; 2 Division of Pathology and Cell Therapy, Chiba Cancer Center Research Institute, Chiba, Japan; 3 Department of Molecular Biology and Oncology, Graduate School of Medicine, Chiba University, Chiba, Japan; 4 Department of Thoracic Disease, Chiba Cancer Center, Chiba, Japan; 5 Department of Respirology, Graduate School of Medicine, Chiba University, Chiba, Japan; 6 Department of Medical Oncology, Graduate School of Medicine, Chiba University, Chiba, Japan; 7 Department of Surgery, School of Medicine, Toho University, Tokyo, Japan; 8 Department of Pathology, Tokyo Women's Medical University Yachiyo Medical Center, Yachiyo, Japan; Faculté de médecine de Nantes, France

## Abstract

We examined anti-tumor effects of zoledronic acid (ZOL), one of the bisphosphonates agents clinically used for preventing loss of bone mass, on human mesothelioma cells bearing the wild-type *p53* gene. ZOL-treated cells showed activation of caspase-3/7, -8 and -9, and increased sub-G1 phase fractions. A combinatory use of ZOL and cisplatin (CDDP), one of the first-line anti-cancer agents for mesothelioma, synergistically or additively produced the cytotoxicity on mesothelioma cells. Moreover, the combination achieved greater anti-tumor effects on mesothelioma developed in the pleural cavity than administration of either ZOL or CDDP alone. ZOL-treated cells as well as CDDP-treated cells induced p53 phosphorylation at Ser 15, a marker of p53 activation, and up-regulated p53 protein expression levels. Down-regulation of p53 levels with siRNA however did not influence the ZOL-mediated cytotoxicity but negated the combinatory effects by ZOL and CDDP. In addition, ZOL treatments augmented cytotoxicity of adenoviruses expressing the *p53* gene on mesothelioma. These data demonstrated that ZOL-mediated augmentation of p53, which was not linked with ZOL-induced cytotoxicity, played a role in the combinatory effects with a p53 up-regulating agent, and suggests a possible clinical use of ZOL to mesothelioma with anti-cancer agents.

## Introduction

The majority of mesothelioma development is tightly linked with occupational asbestos exposure and the patient numbers are increasing worldwide [1], [2]. Approximately 70–80% of mesothelioma cells have the wild-type *p53* gene but show a homologous deletion at the INK4A/ARF locus containing the *p14^ARF^* and the *p16^INK4A^* genes, which consequently leads to decreased p53 functions despite the wild-type genotype [3]–[5]. Prognosis of the mesothelioma patients is dim in most of the cases [1], [2], [6]. Extrapleural pneumonectomy is applicable only for the patients in an early clinical stage and mesothelioma is essentially resistant to radiation. Chemotherapy is therefore the primary treatment but produced limited anti-tumor effects. A combination of cisplatin (CDDP) and pemetrexed is currently the first-line regimen but an average survival period with the agents is about 12 months [7]. The clinical outcome even with the updated combinatory chemotherapy is thus unsatisfactory and a possible second-line agent has not yet been known. A novel therapeutics is thereby required and restoration of decreased p53 functions is one of the strategies.

Bisphosphonates (BPs) are synthetic analogues of pyrophosphate and have a strong affinity for mineralized bone matrix [8]. BPs inhibit bone absorption through interfering osteoclasts' actions, and are currently used as a therapeutic agent for osteoporosis, malignancy-linked hypercalcemia and similar bone diseases. Recent reports demonstrated that BPs also achieved cytotoxicity on tumor cells through apoptosis induction and produced anti-tumor effects *in vitro* [9]. The BPs-mediated effects *in vivo* were evidenced with osseous tumors or with bone metastasis of non-osseous tumors [10]. Moreover, a number of studies also demonstrated the anti-tumor effects *in vivo* with non-osseous tumors despite BPs being readily excreted from body and accumulated in bone tissues [11], [12]. The mechanism of BPs-mediated cytotoxicity is dependent on BPs structures [8], [9]. The first generation of BPs is converted into non-hydrolyzable cytotoxic ATP analogues which decrease mitochondrial membrane potentials. Both the second and the third generations inhibit farnesyl pyrophosphate synthetase and deplete isoprenoid pools, which subsequently results in decreased prenylation of small guanine-nucleotide-binding regulatory proteins (small G proteins). The unprenylated form does not bind to cell membrane and the decreased membrane-bound fraction reduces functions of small G proteins since membrane binding is required for the biological activities including cell survival. It remains however uncharacterized as to the precise mechanisms of cytotoxicity induced by down-regulated functions of small G proteins.

In the present study, we examined cytotoxic activities of zoledronic acid (ZOL), one of the third generation of BPs, on human mesothelioma cells and investigated a possible combinatory use of CDDP with ZOL. We found that ZOL induced up-regulation of p53 expression and the phosphorylation, but down-regulated p53 expression had little effects on the ZOL-induced cytotoxicity. Nevertheless, the ZOL-mediated p53 activation contributed to combinatory effects with CDDP.

## Materials and Methods

### Cells and mice

Human mesothelioma MSTO-211H cells were purchased from American Type Culture Collection (Manassas, VA, USA) and EHMES-10 cells were kindly provided by Dr. Hamada (Ehime Univ., Ehime, Japan) [13]. Expressions of p14^ARF^ and p16^INK4A^ were negative and the *p53* status was wild-type in both cells. BALB/c *nu/nu* mice (6-week-old females) were purchased from Japan SLC (Hamamatsu, Japan).

### Adenoviruses (Ad) preparation

Replication-incompetent type 5 Ad expressing the wild-type *p53* gene (Ad-p53) or the *β-galactosidase* gene (Ad-LacZ), in which the cytomegalovirus promoter activated transcription of the transgene, were prepared with an Adeno-X expression vector system (Takara, Shiga, Japan). The amounts of Ad were expressed as viral particles (vp).

### Cell viability test

Cell viabilities were assessed with a WST reagent (Dojindo, Kumamoto, Japan) by detecting the amounts of formazan produced with absorbance at 450 nm (WST assay). The relative viability was calculated based on the absorbance without any treatments. Half maximal inhibitory concentration (IC_50_) and combination index (CI) values at the fraction affected (Fa) which showed relative suppression levels of cell viability were calculated with CalcuSyn software (Biosoft, Cambridge, UK). Fa = 1 and Fa = 0 indicate 0% and 100% viability assayed with the WST assay, respectively, and CI<1, CI = 1 and CI>1 indicate synergistic, additive and antagonistic actions, respectively.

### Cell cycle

Cells were fixed with 100% ethanol, treated with RNase A (50 µg/ml) for 15 min, and stained with propidium iodide (PI) (50 µg/ml). The fluorescence intensity was analyzed with FACSCalibur and CellQuest software (BD Biosciences, San Jose, CA, USA).

### Caspase activity

Cells treated with ZOL (Novartis Pharmaceuticals, Tokyo, Japan) were tested for the activity of caspase-3/7, -8 or -9 with respective Caspase-Glo kits (Promega, Madison, WI, USA). The relative activity level was calculated based on luminescence intensity of cells without any treatments.

### Western blot analysis

Cell lysate was subjected to sodium dodecyl sulfate-polyacrylamide gel electrophoresis and then transferred to a nitrocellulose membrane, which was further hybridized with antibody (Ab) against p53 (Thermo Fisher Scientific, Fremont, CA, USA), phosphorylated p53 at serine (Ser) residue 15 (Cell Signaling, Danvers, MA, USA), unprenylated Rap1A (Santa Cruz Biotechnology, Santa Cruz, CA, USA) or actin (Sigma-Aldrich, St Louis, MO, USA) as a control, followed by an appropriate second Ab. The membranes were developed with the ECL system (GE Healthcare, Buckinghamshire, UK).

### RNA interference

Cells were transfected with small interfering RNA (siRNA) duplex targeting p53 or with non-coding siRNA as a control (Invitrogen, Carlsbad, CA, USA) for 24 h using Lipofectamine RNAiMAX according to the manufacturer's protocol (Invitrogen).

### Animal experiments

MSTO-211H cells were injected into the pleural cavity of BALB/c *nu/nu* mice. ZOL (25 µg) or the same amount of phosphate-buffered saline (PBS) was administrated intrapleurally on day 3, and CDDP (Bristol-Myers Squibb, New York, USA) (100 µg) or the same amount of PBS was injected intraperitoneally on day 5. In this animal model, tumors became visible on day 9. The mice were sacrificed on day 24 and the tumor weights were measured. The animal experiments were approved by the animal experiment and welfare committee at Chiba University and were performed according to the guideline on animal experiments.

## Results

### ZOL-induced cytotoxicity and caspase activation

**Figure 1:**
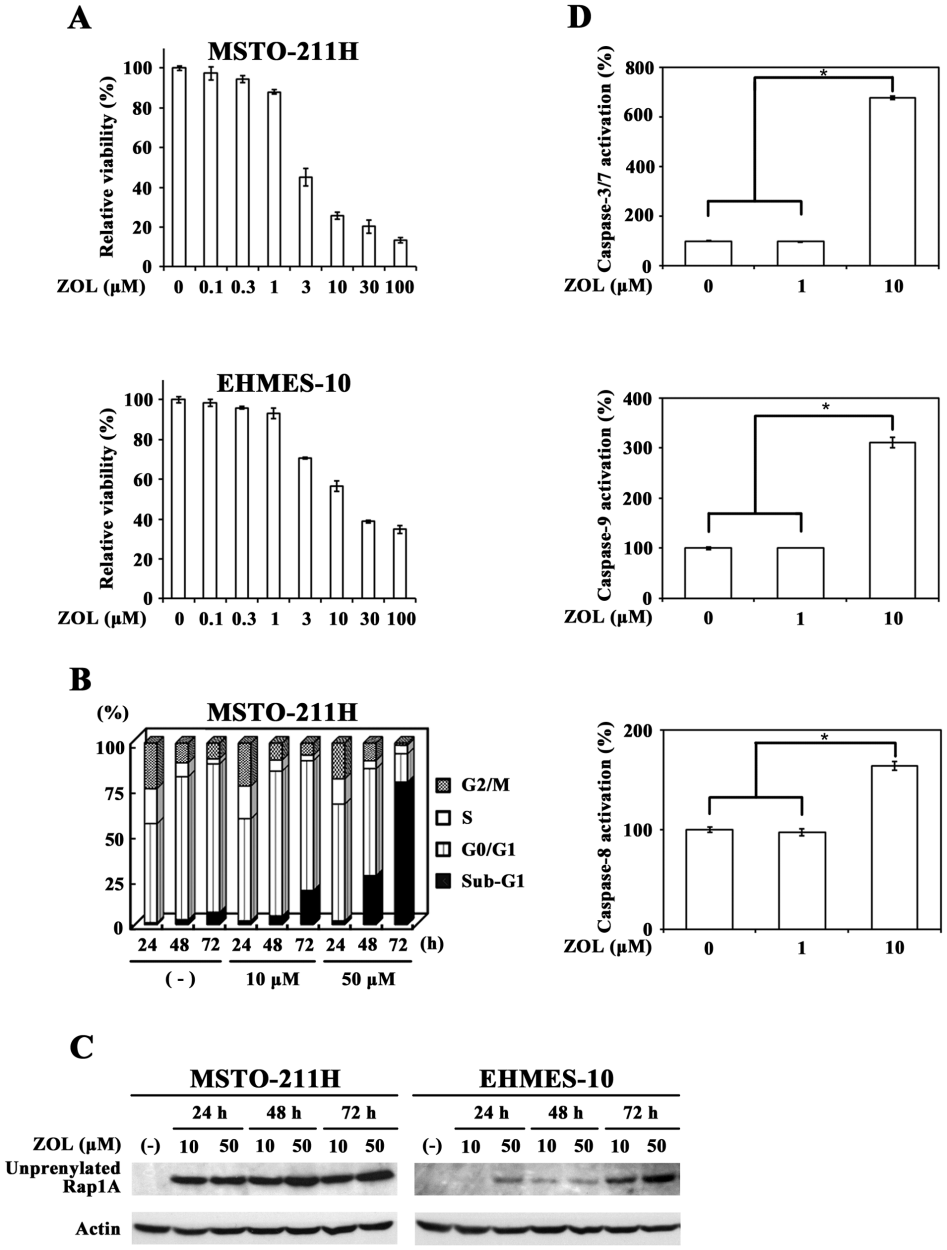
ZOL-induced cytotoxicity to mesothelioma. (A) Cells were treated with different concentrations of ZOL for 3 days and the cell viabilities were measured with the WST assay. Means of triplicated samples and the SD bars are shown. (B) Flow cytometrical analyses of cell cycle progression in ZOL-treated MSTO-211H cells. (C) Western blot analyses of unpreylated Rap1A expressions in cells treated with ZOL. Actin was used as a loading control. (D) Caspase activations in MSTO-211H cells that were treated with ZOL for 3 days were assayed with respective luminescence-based kits. The activities of untreated cells were expressed as 100%. Means of triplicated samples and the SE bars are shown. * P<0.01.

We examined a possible cytotoxic action of ZOL on mesothelioma cells with the WST assay and found that both mesothelioma cells, MSTO-211H and EHMES-10, were susceptible to ZOL with a dose-dependent manner (Fig. 1A). Cell cycle analyses showed that ZOL increased sub-G1 phase fractions in MSTO-211H cells (Fig. 1B, Table 1), indicating that ZOL induced cell death. We also tested ZOL-induced unprenylation of Rap 1A, one of small G proteins, and confirmed that ZOL inhibited the prenylation in both cells (Fig. 1C). We investigated a possible activation of caspase-3/7, -9 and -8 by testing the cleaving activity of a specific substrate (Fig. 1D). ZOL treatments at 1 µM did not induce activation of respective caspases but those at 10 µM activated the caspases in MSTO-211H cells. These data collectively indicated that ZOL treatments activated cell death processes through the caspase cleavages in mesothelioma cells.

**Table 1:** Cell cycle distribution of ZOL-treated cells.

		Cell cycle distribution (% ± SE)			
ZOL (Concentration)	Time	Sub-G1	G0/G1	S	G2/M
(-)	24 h	1.00±0.08	54.83±0.46	19.34±0.17	25.18±0.37
(-)	48 h	2.66±0.10	78.68±0.27	7.68±0.27	10.82±0.12
(-)	72 h	6.83±0.15	82.23±0.29	2.87±0.16	8.68±0.07
10 µM	24 h	2.12±0.10	56.88±0.33	18.19±0.28	23.90±0.24
10 µM	48 h	4.75±0.13	80.28±0.13	6.13±0.19	9.38±0.14
10 µM	72 h	18.84±0.12	71.53±0.21	3.09±0.03	6.58±0.14
50 µM	24 h	2.01±0.16	64.58±0.11	13.97±0.18	19.78±0.11
50 µM	48 h	26.98±0.76	59.05±0.53	4.37±0.21	9.70±0.07
50 µM	72 h	79.14±0.32	15.65±0.13	4.73±0.06	1.22±0.11

MSTO-211H cells were treated with or without ZOL (at 10 or 50 µM) for 24–72 h. Cell cycle was analyzed with flow cytometry.

### Combinatory cytotoxic effects of ZOL and CDDP

**Figure 2:**
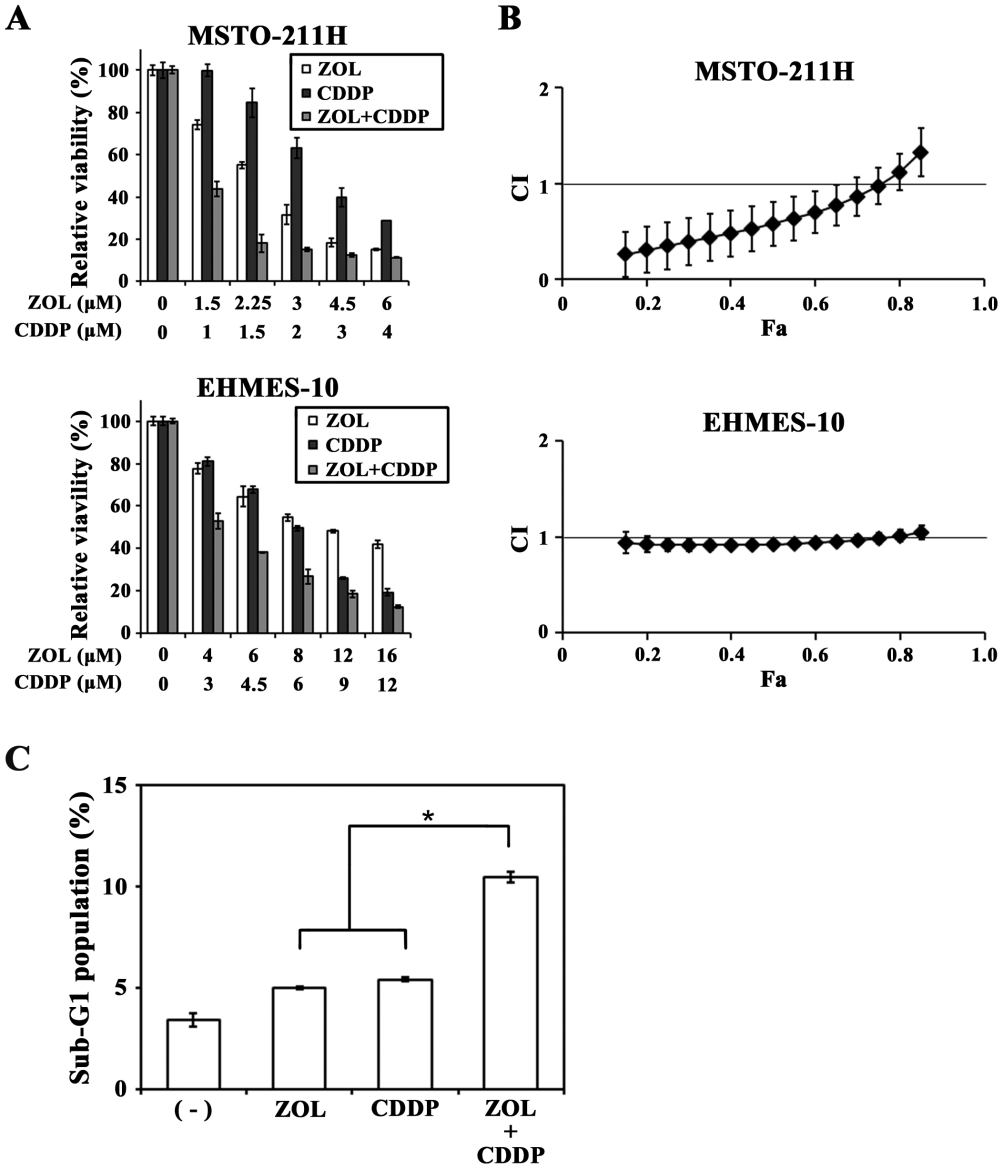
Combinatory effects of ZOL and CDDP. (A) Cells were treated with different doses of ZOL and CDDP at a constant concentration ratio (ZOL∶CDDP = 3∶2 at each concentration in MSTO-211H and 4∶3 in EHMES-10 cells) for 3 days and the cell viabilities were measured with the WST assay. Means of triplicated samples and the SD bars are shown. (B) CI values based on the cell viabilities as shown in (A) were calculated at different Fa points with CalcuSyn software. The SE bars are also indicated. (C) Sub-G1 phase populations of PI-stained MSTO-211H cells that were treated with ZOL (15 µM) and/or CDDP (4 µM) for 24 h were calculated with flow cytometry. Means of triplicated samples and the SE bars are shown. * P<0.01.

We investigated combinatory effects of ZOL and CDDP in MSTO-211H and EHMES-10 cells. We calculated respective IC_50_ values of each agent to know an optimal test rang and then examined cytotoxicity at various doses of both agents with a constant concentration ratio according to the CalcuSyn software instruction. Combination of ZOL and CDDP achieved cytotoxicity greater than each agent (Fig. 2A) and statistical analyses showed that CI values at Fa points below 0.8 in MSTO-211H cells were less than 1 and those between 0.15 and 0.8 Fa points in EHMES-10 cells were close to but under 1 (Fig. 2B). These CI values demonstrated that both ZOL and CDDP achieved cytotoxicity synergistically in MSTO-211H cells, and additively, or possibly slightly synergistically, in EHMES-10 cells. Cell cycle analyses indicated that sub-G1 phase populations in ZOL- plus CDDP-treated MSTO-211H cells were greater than those in ZOL- or CDDP- treated cells (Fig. 2C), suggesting that the enhanced cytotoxic activities by the combination of ZOL and CDDP were attributable to increased apoptotic cell death.

### Combinatory effects of ZOL and CDDP *in vivo*

**Figure 3:**
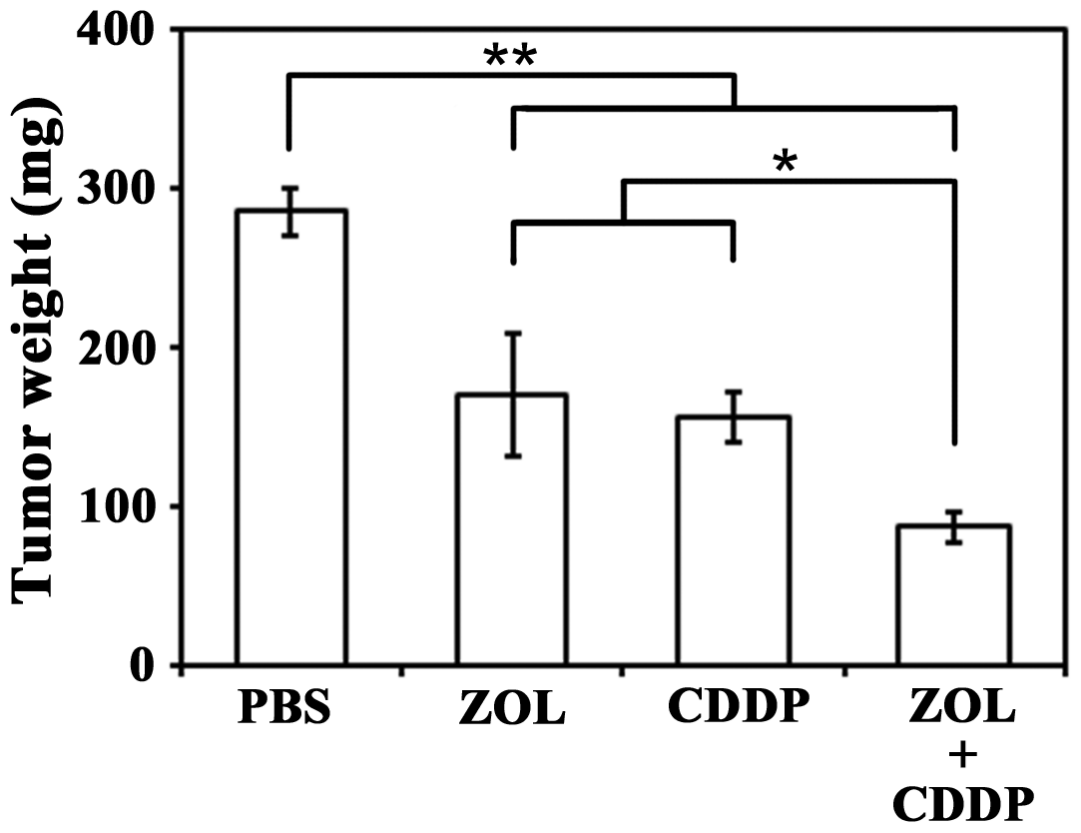
Combinatory effects with ZOL and CDDP in an orthotopic animal model. MSTO-211H cells (1×10^6^) were inoculated into the pleural cavity of BALB/c *nu/nu* mice (n = 6) (day 1), and then ZOL (25 µg, day 3) was administrated into the pleural cavity and/or CDDP (100 µg, day 5) into the peritoneal cavity (CDDP). PBS was used as a control. Tumor weights were measured on day 24. The SE bars are also shown. * P<0.05, ** P<0.01.

We investigated anti-tumor effects of ZOL in combination with CDDP in an orthotopic animal model (Fig. 3). Nude mice injected with MSTO-211H cells in the pleural cavity received ZOL intrapleurally and/or CDDP intraperitoneally. All the tumors were found in the pleural cavity without any detectable extrapleural metastatic foci. ZOL or CDDP administration inhibited the tumor growth compared with PBS-injected group. A combinatory administration of ZOL and CDDP further decreased tumor weights, demonstrating that the combination produced greater therapeutic effects than the case treated with a single agent. We did not notice body weight loss in the combinatory group, indicating that the combination was not toxic to the tested animals.

### ZOL induced p53 activation

**Figure 4:**
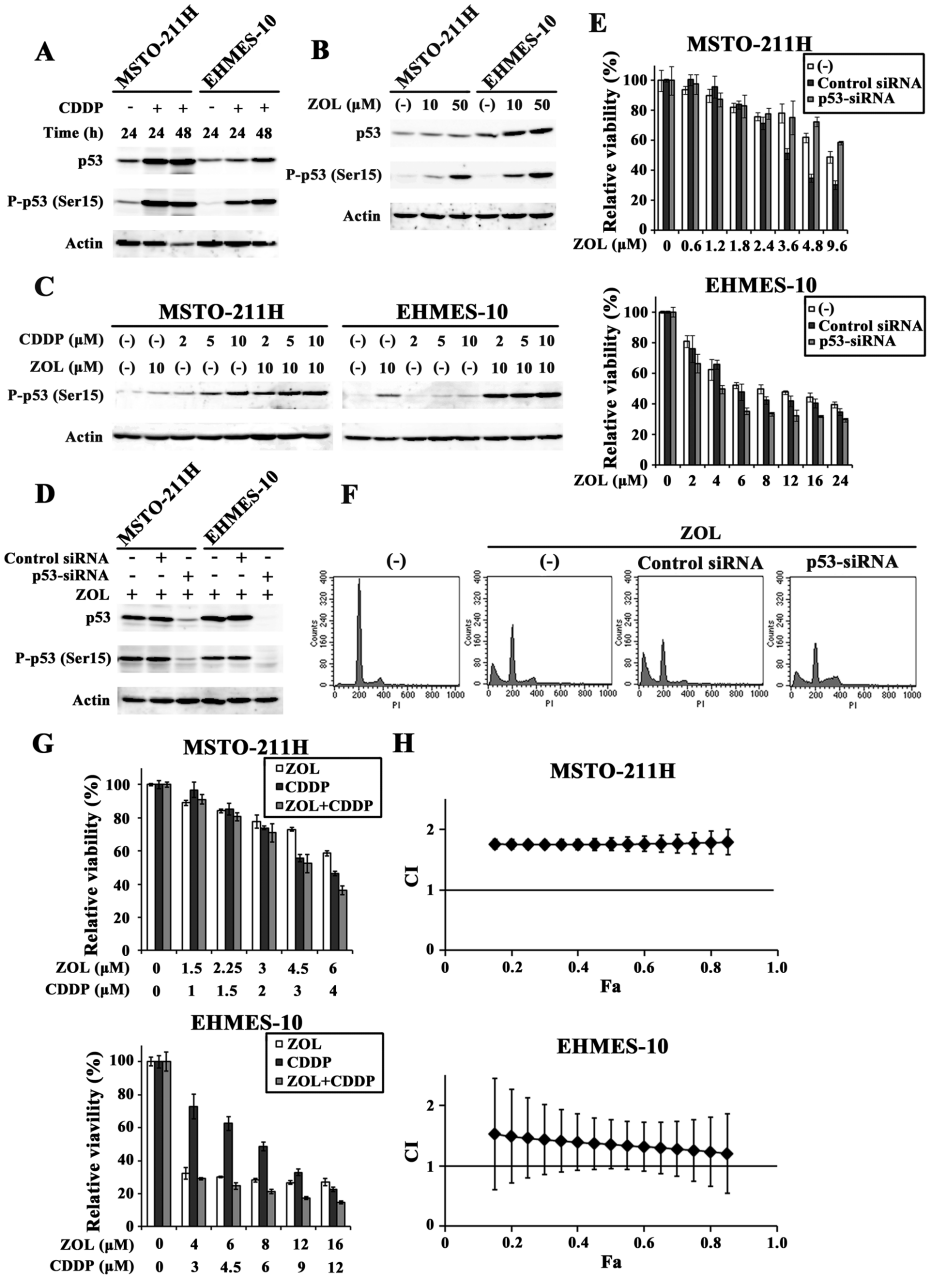
ZOL-induced up-regulation of p53 and knockdown of the p53 expressions with siRNA. (A, B) CDDP-treated (20 µM) and ZOL-treated (48 h) cells were subjected to Western blot analysis and probed with antibodies as indicated. Actin was used as a loading control. (C) Cells were treated with CDDP and/or ZOL for 48 h at the indicated concentrations and the expression levels of phosphorylated p53 were examined. (D) Cells were transfected with p53-targeted siRNA (p53-siRNA) or non-targeted control siRNA (Control) for 24 h and then treated with ZOL (50 µM) for 48 h. The lysate was subjected to Western blot analysis. (E) Cells were transfected with siRNA as indicted and were treated with ZOL for 3 days. The cell viabilities were measured with the WST assay and means of triplicated samples with the SD bars are shown. (F) Flow cytometrical analyses of MSTO-211H cells that were transfected with respective siRNA for 24 h and then treated with ZOL (50 µM) for 48 h. (G, H) Cells transfected with p53-siRNA were treated with different doses of ZOL and CDDP as indicated for 3 days and the CI values based on the cell viabilities were calculated at different Fa points with CalcuSyn software.

We examined whether p53 activation was involved in the ZOL-mediated cytotoxicity since the p53 pathways play a key role in apoptosis induction. Firstly, we tested possible p53 activation in wild-type *p53* mesothelioma with CDDP (Fig. 4A). CDDP-treated MSTO-211H and EHMES-10 cells induced phosphorylation of p53 at the Ser 15 residue, a hallmark of p53 activation, and up-regulated p53 protein levels. We then examined influence of ZOL on p53 expressions and found that ZOL treatments phosphorylated p53 at Ser 15 and augmented p53 protein levels in both cells (Fig. 4B). These data showed that ZOL induced p53 activation and subsequently raised a possibility that the ZOL-mediated cytotoxicity was caused by p53 activation. We also investigated the combinatory effects of CDDP and ZOL on the p53 phosphorylation at Ser 15 (Fig. 4C). The phosphorylation level in cells treated with both agents was greater than that in cells treated with either CDDP or ZOL, suggesting that both agents cooperatively activated the p53 pathways.

### Down-regulated p53 action on cytotoxicity and on combination effect

We further investigated a possible involvement of p53 activation in the ZOL-mediated cytotoxicity by down-regulating p53 expression with siRNA. The p53-siRNA treatment markedly decreased p53 expression and the phosphorylation level (Fig. 4D). The down-regulated p53 however minimally affected the ZOL-induced cytotoxicity in MSTO-211H cells, at least in lower concentrations, and rather slightly enhanced the cytotoxicity in EHMES-10 cells (Fig. 4E). Control siRNA treatments unexpectedly increased the cytotoxicity in MSTO-211H cells at high ZOL concentrations. These data suggested that the ZOL-mediated cytotoxicity was independent of p53 activation. We also analyzed cell cycle changes in ZOL-treated MSTO-211H cells after they were transfected with p53-siRNA (Fig. 4F, Table 2). Cell cycle distributions showed that p53-siRNA treatments marginally influenced the ZOL-mediated increase of sub-G1 phase populations. The decreased level of sub-G1 phase fractions due to the p53-siRNA treatment was disproportionately lower than that of the p53 protein expression after transfection with siRNA. In contract, the p53-siRNA treatment increased S and G2/M phase and decreased G0/G1 phase fractions, showing that down-regulated p53 promoted cell cycle progression. These data demonstrated that decreased p53 levels influenced the cell cycle but little affected the ZOL-mediated cytotoxicity, and confirmed that the ZOL-induced p53 activation was irrelevant to the ZOL-mediated cytotoxicity. Control-siRNA treated cells increased sub-G1 phase fractions, which accorded with the WST results. It could be due to non-specific cytotoxicity of control siRNA in MSTO-211H cells but the mechanism underling is currently unknown.

**Table 2:** Cell cycle distribution of p53-siRNA-treated cells.

		Cell cycle distribution (% ± SE)			
siRNA for	ZOL	Sub-G1	G0/G1	S	G2/M
(−)	(−)	2.35±0.07	81.69±0.36	6.88±0.29	8.79±0.33
(−)	(+)	34.53±0.23	50.39±0.13	6.12±0.11	8.32±0.29
Control	(+)	52.34±0.60	38.23±0.32	3.79±0.08	5.10±0.27
p53	(+)	28.36±0.12	38.59±0.16	16.69±0.17	15.53±0.17

MSTO-211H cells were transfected with or without siRNA for 24 h, and then treated with or without 50 µM ZOL for further 48 h. Cell cycle was analyzed with flow cytometry.

We also examined whether the combinatory effects of ZOL and CDDP were modulated by p53 expression levels (Fig. 4G and H). The p53-siRNA treatments nullified the synergistic or the additive effects detected in MSTO-211H and EHMES-10 cells. The CI values of the combination under the p53-siRNA treatments were more than 1, which indicated rather antagonistic actions. Activation of p53 was thus involved in the combinatory effects of ZOL and CDDP although it was not related with the ZOL-mediated cytotoxicity.

### Combinatory effects of ZOL and Ad-p53

**Figure 5:**
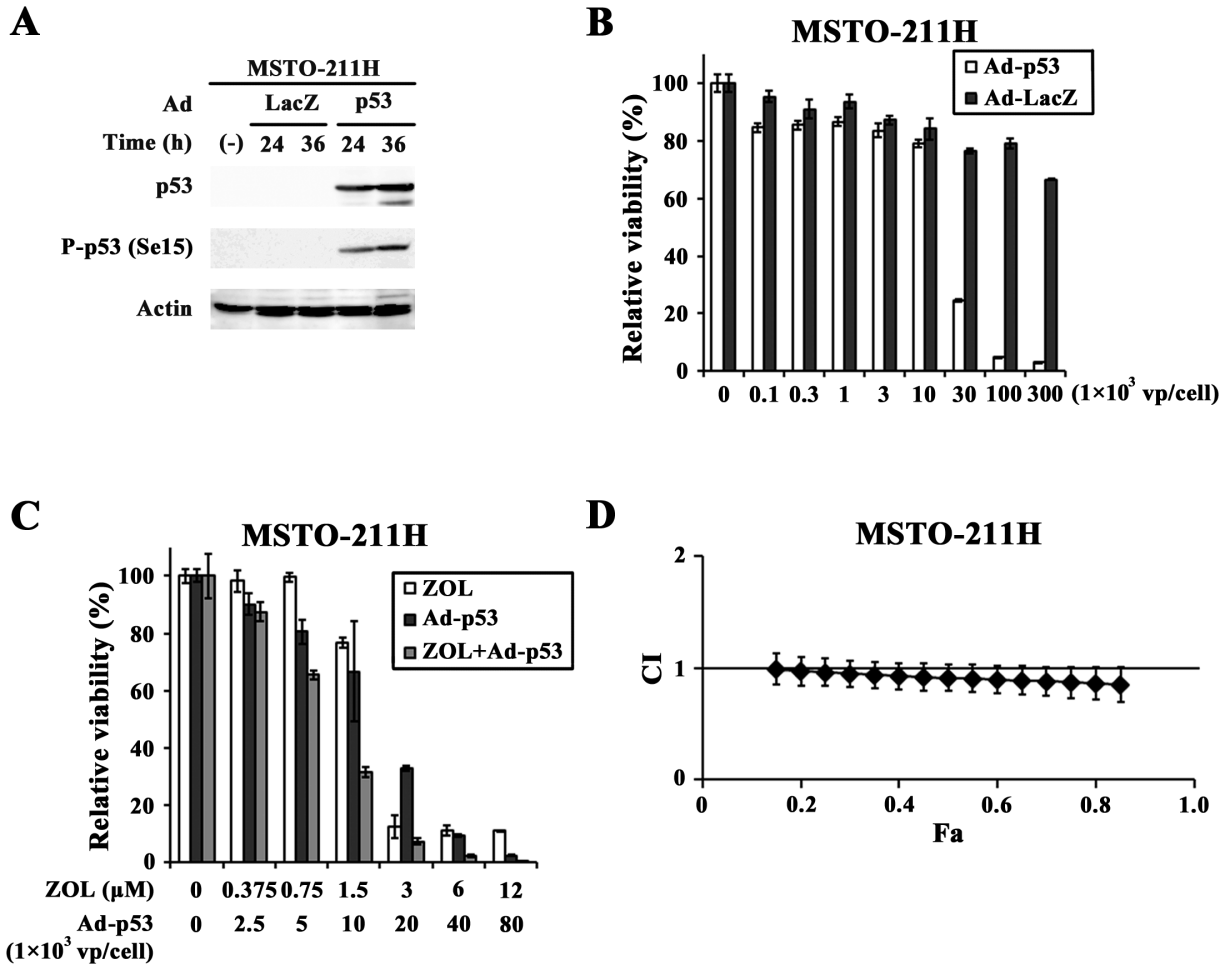
Combinatory effects with ZOL and Ad-p53. (A) Cells were infected with Ad-p53 or Ad-LacZ (1×10^3^ vp/cell) as a control and were subjected to Western blot analysis. Actin was used as a loading control. (B) Cells were infected with Ad-p53 or Ad-LacZ and the cell viabilities were measured with the WST assay. Means of triplicated samples and the SD bars are shown. (C, D) Cells were infected with Ad-p53 and/or treated with ZOL as indicated and cultured for 3 days. The cell viabilities were measured with the WST assay and CI values based on the cell viabilities were calculated at different Fa points with CalcuSyn software.

We examined whether up-regulated p53 levels by ZOL increased p53-mediated cytotoxicity. Transduction of MSTO-211H cells with Ad-p53 but not Ad-LacZ increased p53 expressions and induced the phosphorylation at Ser 15 (Fig. 5A). Moreover, Ad-p53 but not Ad-LacZ decreased the cell viability with a dose-dependent manner (Fig. 5B), demonstrating that induction of p53 produced cytotoxic effects in MSTO-211H cells. We then examined combinatory effects of Ad-p53 and ZOL at a constant ratio between the agents (Fig. 5C). The combination produced additive, or possibly slightly synergistic, effects at above 0.15 Fa points. (Fig. 5D) and suggested that up-regulation of p53 by ZOL enhanced Ad-p53-mediated cytotoxicity by further activating the p53 pathways.

## Discussion

In this study we demonstrated that ZOL alone and the combination with CDDP produced anti-tumor effects on mesothelioma. ZOL up-regulated p53 expression but the ZOL-mediated cytotoxicity was scarcely dependent on the p53 induction, suggesting that the cytotoxicity was due to inhibition of small G proteins' functions. Down-regulated p53 levels on the other hand negated the synergistic actions by ZOL and CDDP, indicating that the ZOL-induced p53 activation contributed to the combinatory anti-tumor effects produced with CDDP.

The majority of mesothelioma cells has defect of p14^ARF^, which results in an increased level of Mdm2 that induces p53 degradation [14], [15]. Augmentation of p53 is therefore a possible therapeutic strategy for mesothelioma by restoring p53 functions [16]. The present study indicated that ZOL phosphorylated p53 and up-regulated the expression levels, suggesting a crucial role of p53 induction in the ZOL-mediated cytotoxicity. ZOL in fact activated caspases and increased sub-G1 phase populations. The knockdown experiments with p53-siRNA however demonstrated that p53 activation itself did not contribute to the ZOL-mediated cytotoxic actions. A possible involvement of the p53 pathways in ZOL-mediated cytotoxicity may need further investigations but the present data evidenced that the up-regulated p53 level in ZOL-treated cells was irrelevant to the cytotoxicity as reported previously [17], [18]. The ZOL-induced cytotoxicity can be therefore attributable to inhibited prenylation of small G proteins [8]–[10].

ZOL-induced activation of p53 nevertheless contributed to the cytotoxicity by other agents of which the functions were linked with p53 levels. CDDP is one of such agents and augmented p53 levels in target tumors facilitate CDDP-induced cell death [19], [20]. In fact our previous study showed that Ad-p53-transduced MSTO-211H cells produced synergistic cytotoxicity with CDDP, and that the CI values were below 1 between 0.2 and 0.8 Fa points [21]. The present study demonstrated that combination of ZOL and CDDP produced synergistic or additive anti-tumor effects on mesothelioma with the wild-type *p53* gene. The combination increased sub-G1 phase populations and decreased tumor volumes in an orthotopic animal model, but down-regulation of p53 with the siRNA completely nullified the combinatory effects. These data suggested that ZOL-induced p53 up-regulation favored CDDP-mediated cytotoxicity through further augmenting the p53 pathways. Benassi *et al* recently reported similar results with paired cells, *p53*-mutated and the isogeneic *p53*-wild-type parent cells from osteosarcoma, that combinatory effects of ZOL and CDDP were p53-dependent [20]. The present study furthermore analyzed the interactions between the two agents and demonstrated synergistic or additive actions in the combination as well as the *in vivo* efficacy. The interactions became antagonistic under the p53-siRNA treatment, which suggested that loss of ZOL-induced p53 up-regulation was rather inhibitory to CDDP-mediated cytotoxicity. These data consequently suggest that the ZOL-mediated up-regulated p53 pathways contributed to combinatory effects with CDDP. ZOL-mediated inhibitory actions on small G proteins' prenylation were probably not influenced by cellular p53 levels because down-regulation of p53 did not affect the ZOL-mediated cytotoxicity. The inhibited prenylation itself may produce possible combinatory effects with CDDP but the p53-siRNA treatment which produced antagonistic effects suggested that mechanistic association between unprenylated small G proteins and CDDP was unlikely.

Transduction levels of Ad-p53 determined p53-dependent cytotoxicity, and a combinatory use of ZOL and Ad-p53 produced additive, and possibly slightly synergistic, cytotoxic effects. A possible role of Ad-p53 in the combinatory effects through inducing further unpreylation of small G proteins was probably minimal since ZOL-mediated cytotoxicity was independent of p53 levels. Nevertheless, ZOL augmented endogenous p53 levels and the up-regulation appeared to sensitized tumor cells to be susceptible to a p53 up-regulating agent. ZOL can induce unprenylation of non-small G proteins but it remains uncharacterized whether such unprenylated non-small G proteins can produce cytotoxicity in ZOL-treated cells. Synergism between CDDP and ZOL was greater than that between Ad-p53 and ZOL probably because CDDP-mediated p53 up-regulation and over-expression of p53 with Ad-p53 are not equal from the standpoint of signal transduction systems. For example, CDDP-treated cells can activate non-p53-mediated pathways and Ad-mediated transduction activates type I interferons-mediated pathways.

The present data suggested a possible clinical application of ZOL for mesothelioma in combination with CDDP or Ad-p53. In fact, Ad-p53 has been used in clinical trials [22], and ZOL and CDDP are commonly used for cancer patients [8], [23]. We demonstrated combinatory anti-tumor effects of ZOL and CDDP on non-osseous tumors as reported on osseous tumors [20], [24]. Therapeutic activities of ZOL on tumors nevertheless seem to be less significant in non-osseous tissues than those in osseous tissues [9], [10] because ZOL is readily excreted from kidney and cannot be maintained at a high concentration except in bone tissues [10], [11]. Recent studies however showed that ZOL in combination with imatinib and doxorubicin produced greater cytotoxicity than monotherapy even against non-osseous tumors, Bcr-Abl-positive leukemina [25] and breast cancer [26], respectively. These data indicated that ZOL, even through a systemic administration route, produced anti-tumor effects together with other cytotoxic agents. Moreover, mesothelioma can be one of the suitable targets of ZOL in clinical settings because the intrapleural administration is speculated to keep a relative high concentration of ZOL at tumor sites compared with an intravenous injection, although this remains to be proven. The present study suggests that ZOL administered intrapleurally and CDDP injected systemically may produce a therapeutic benefit to mesothelioma patients. Our preliminary study showed that intrapleural administration of 40 µg ZOL at a concentration of 0.4 mg/ml in mice, which was equivalent to 7.8–9.8 mg in human [27] and was 10 times higher drug concentration than the current clinical dose (4 mg in total and 0.04 mg/ml at the concentration), did not cause any body weight changes or other adverse reactions such as inflammatory reactions (data not shown), showing a feasible intrapleural injection of ZOL with safe.

We also showed that Ad-p53 suppressed the viability of mesothelioma and produced combinatory anti-tumor effects with ZOL. Intrapleural injections of Ad-p53 were in fact conducted safely in patients with pleural effusions [28]. Previous studies demonstrated that Ad-p53 activated the p53 pathways and achieved combinatory anti-tumor effects with an anti-cancer agent including CDDP [21], [29], [30]. The mechanism of ZOL-mediated p53 induction remains unclear but the p53-inducible p21 is a downstream target of Ras and RhoA, the major molecules of small G proteins [31]. Inhibited protein prenylation can cause downstream activation of p53, and ZOL thereby is a candidate to analyze a possible cross-talk between small G proteins and the p53 pathways. Previous studies also showed that combinatory cytotoxicity of ZOL and an anti-cancer agent was linked with ZOL-mediated inhibition of P-glycoprotein functions [24] and that a combinatory use of doxorubicin and ZOL inhibited angiogenesis [26]. These studies indicated possible p53-independent cytotoxicity of ZOL that could synergize with other agents through multiple mechanisms.

In conclusion, we demonstrated that ZOL produced cytotoxic activities on mesothelioma and a combinatory use with CDDP or Ad-p53 produced better therapeutic effects than monotherapy with a single agent. ZOL-mediated p53 up-regulation was not involved in the ZOL-induced cytotoxicity in EHMES-10 cells, and in MSTO-211H cells at least at low concentrations at which synergistic effects were observed with CDDP, but contributed to combinatory anti-tumor effects of CDDP or Ad-p53. Based on the current study we presume that an intrapleural injection of ZOL, which is technically feasible, in combination with CDDP, the first-line agent for mesothelioma, is a potential therapeutics for mesothelioma.
